# Th17 is involved in the pathogenesis of Bechet's disease via CCL20-CCR6 axis

**DOI:** 10.1186/ar3680

**Published:** 2012-02-09

**Authors:** Hidekata Yasuoka, Zhu Chen, Tsutomu Takeuchi, Masataka Kuwana

**Affiliations:** 1 Department of Internal Medicine, Keio University School of Medicine, Tokyo, 160-8582, Japan; 2 Department of Rheumatology and Immunology, Anhui Provincial Hospital, Hefei, 230001, China

## Background

Behcet's disease (BD) is an autoinflammatory disease with a unique distribution characterized by uveitis, and mucosal and skin lesions, which are characterized by the prominent infiltration of immune cells such as lymphocytes and neutrophils. A novel helper T-cell subset Th17, IL-17-producing helper T cells, has been appreciated [1]. IL-17 is involved in the induction of a series of chemokines, growth factors, proteases, and cytokines, and production of IL-17 results in induction of neutrophil migration and chronic inflammation [2]. Based on these findings, we hypothesized that Th17 is involved in the pathogenesis of BD.

## Materials and methods

To examine a role of Th17 response in the pathogenic process of BD, peripheral blood samples from 20 patients with BD and 14 controls were used to evaluate phenotypic and functional properties relevant to the Th17 response. Plasma IL-17 and CCL20 levels were examined using ELISA. Expression levels of RORC mRNA in CD4^+ ^T cells were examined by RT-PCR and CD4^+ ^cells expressing IL-17, CCR6 was examined by flow cytometry. Evaluation of chemotaxis of CD4^+ ^T cells toward CCL20 was examined by migration assay using TransWell^® ^double chamber system.

## Results

Plasma IL-17 was higher in active BD compared with healthy controls (P < 0.05). Expression levels of RORC mRNA in peripheral blood mononuclear cells by RT-PCR and proportion of CD4^+ ^cells expressing intracellular IL-17 were increased in patients with BD than in controls (P < 0.05 in both comparisons). Expression of chemokine receptor CCR6 was detected in nearly all IL-17-expressing cells. The proportion of CD4^+^CCR6^+ ^was higher in BD patients in remission compared those with active disease (P < 0.05), suggesting that these cells are migrated to the lesions at active disease phase. In addition, CD4^+ ^T cells from BD patients had enhanced migration capacity induced by CCL20, than did those from controls. Finally, CCL20 level was higher in BD patients than in controls (*P *< 0.05).

## Conclusions

These results together suggest that Th17 are involved in the pathogenesis of BD by migrating into the lesions of BD through the CCL20-CCR6 axis.
